# Supplementary material for: Transcriptomic and epigenetic regulation of hair cell regeneration in the mouse utricle and its potentiation by Atoh1
Source: eLife. 2019 Apr 29;8:e44328. doi: 10.7554/eLife.44328 (PMC6504235; doi:10.7554/eLife.44328)
Supplement: Figure 1—source data 1. [file elife-44328-fig1-data1.docx]

| Top 100 utricle hair cell-enriched genes | | | | | | | | | |
| --- | --- | --- | --- | --- | --- | --- | --- | --- | --- |
|  |  |  |  |  |  |  |  |  |  |
| **Ear disease-related** | | **Reported in hair cell expression studies** | | | | | **Not Previously Reported** | | |
| Cabp2  (Schrauwen et al., 2012) | | 1700003M02Rik | Cyp4f39 | Ica1l | Mtmr11 | Sult4a1 | 5730409E04Rik | Gm19557 | Ralyl |
| Cdc14a  (Delmaghani et al., 2016) | | 1700026D08Rik | D630023F18Rik | Insc | Nceh1 | Syt13 | Art1 | Grin2d | Rsph14 |
| Dfnb59  (Ebermann et al., 2007) | | 1700101E01Rik | Dnah5 | Iqcg | Nefl | Tcp11 | Art3 | Ift74 | St6galnac3 |
| Elmod1  (Johnson et al., 2012) | | Apba1 | Dner | Irx2 | Nrsn1 | Tekt2 | Asic1 | Iqca | Synpr |
| Gldn  (Johnson et al., 2012) | | Ap3b2 | E230025N22Rik | Kcnb1 | Nrxn3 | Tmem179 | Cbln4 | Kcnh7 | Trim37 |
| Kcna10  (Lee et al., 2013) | | Acss2 | Fcrlb | Kcnma1 | Ofcc1 | Tmem41a | Cfap43 | Lce6a | Tunar |
| Lhfpl5  (Shabbir et al., 2006) | | Ano3 | Fsip1 | Kifap3 | Pip4k2a | Ttc21a | Cfap45 | Lppr5 | Zfp763 |
| Ocm  (Tong et al., 2016) | | Ap3m2 | Gdap1l1 | Kncn | Rarb | Ugt8a | Cnot8 | Mirc35hg |  |
| Pcdh20  (Vuckovic et al., 2015) | | Cd164l2 | Gm1322 | Lrguk | Rph3al | Zcchc12 | Dhrs3 | Nsg1 |  |
| Pls1  (Taylor et al., 2015) | | Chrna10 | Gm5111 | Lrrc10b | Sncg |  | Dlgap1 | Oosp2 |  |
| Ptprq  (Goodyear et al., 2012) | | Cpne9 | Gm9047 | Lrtm2 | Sorbs1 |  | Elovl4 | Pak3 |  |
| Tomt  (Ahmed et al., 2008) | | Cxcl14 | Hsd17b7 | Mkrn2os | Spag8 |  | Fez1 | Rabl2 |  |

| Top 100 utricle supporting cell-enriched genes | | | | | | | | | | | | | | | | | |
| --- | --- | --- | --- | --- | --- | --- | --- | --- | --- | --- | --- | --- | --- | --- | --- | --- | --- |
|  |  |  |  | |  | |  | |  |  | |  | |  | |  | |
| **Ear disease-related** | | **Reported in hair cell expression studies** | | | | | | | **Not Previously Reported** | | | | | | | | |
| Lrp2  (Knipper et al., 2006) | | 9130008F23Rik | | Gadd45b | | Nes | | Trpm3 | 1810011O10Rik | | Crabp2 | | Ifrd1 | | Plet1 | | Tspo |
| Cpxm2  (Somma et al., 2012) | | Ano6 | | Hbegf | | Psmb9 | | Tspan8 | Abca13 | | Ddah2 | | Kazald1 | | Plin3 | | Tubb6 |
| Pttg1  (Lubka-Pathak et al., 2011) | | Anxa1 | | Hspb2 | | Pvr | |  | Aldoc | | Dmd | | Lxn | | Prdx4 | | Wwtr1 |
| Dclk1  (Girotto et al., 2014) | | Capns1 | | Kcnk1 | | Rbp1 | |  | Apoc1 | | Efna1 | | Mest | | Ptms | | Zfp36 |
| Sox10  (Pingault et al., 2015) | | Cdh1 | | Krt18 | | Rhpn2 | |  | Arhgef26 | | F3 | | Mfsd2a | | Rbm47 | | Zfp36l1 |
| Actb  (Perrin et al., 2010) | | Cldn3 | | Krt8 | | Rnase1 | |  | Arpc1b | | Fkbp9 | | Mpzl2 | | Renbp | |  |
| Ddit3  (Fujinami et al., 2012) | | Cldn4 | | Lamc2 | | Sorl1 | |  | Cd63 | | Fosb | | Mtap | | Rhoc | |  |
| Otog  (Goodyear & Richardson, 2002) | | Cnn2 | | Mal | | Sox9 | |  | Ces1d | | Gng11 | | Nupr1 | | Rhou | |  |
| Tecta  (Yamamoto et al., 2017) | | Ctsh | | Mbnl2 | | Sparc | |  | Chpt1 | | Gstm2 | | Pdk4 | | Siae | |  |
|  | | Cyp2d22 | | Mcl1 | | Tes | |  | Cited1 | | H2-K1 | | Pea15a | | Slc44a3 | |  |
|  |  | Fam101b | | Mfi2 | | Thbs1 | |  | Clic1 | | H2-T23 | | Penk | | Timp3 | |  |
|  |  | Fcgrt | | Muc15 | | Tm4sf1 | |  | Coq10b | | Hk2 | | Plaur | | Tspan7 | |  |

**References**

Ahmed, Z.M., Masmoudi, S., Kalay, E., Belyantseva, I.A., Mosrati, M.A., Collin, R.W., Riazuddin, S., Hmani-Aifa, M., Venselaar, H., Kawar, M.N., Tlili, A., van der Zwaag, B., Khan, S.Y., Ayadi, L., Riazuddin, S.A., Morell, R.J., Griffith, A.J., Charfedine, I., Caylan, R., Oostrik, J., Karaguzel, A., Ghorbel, A., Riazuddin, S., Friedman, T.B., Ayadi, H., Kremer, H., 2008. Mutations of LRTOMT, a fusion gene with alternative reading frames, cause nonsyndromic deafness in humans. Nat Genet 40, 1335-1340.

Delmaghani, S., Aghaie, A., Bouyacoub, Y., El Hachmi, H., Bonnet, C., Riahi, Z., Chardenoux, S., Perfettini, I., Hardelin, J.P., Houmeida, A., Herbomel, P., Petit, C., 2016. Mutations in CDC14A, Encoding a Protein Phosphatase Involved in Hair Cell Ciliogenesis, Cause Autosomal-Recessive Severe to Profound Deafness. Am J Hum Genet 98, 1266-1270.

Ebermann, I., Walger, M., Scholl, H.P., Charbel Issa, P., Luke, C., Nurnberg, G., Lang-Roth, R., Becker, C., Nurnberg, P., Bolz, H.J., 2007. Truncating mutation of the DFNB59 gene causes cochlear hearing impairment and central vestibular dysfunction. Hum Mutat 28, 571-577.

Fujinami, Y., Mutai, H., Mizutari, K., Nakagawa, S., Matsunaga, T., 2012. A novel animal model of hearing loss caused by acute endoplasmic reticulum stress in the cochlea. Journal of pharmacological sciences 118, 363-372.

Girotto, G., Vuckovic, D., Buniello, A., Lorente-Canovas, B., Lewis, M., Gasparini, P., Steel, K.P., 2014. Expression and replication studies to identify new candidate genes involved in normal hearing function. PLoS One 9, e85352.

Goodyear, R.J., Jones, S.M., Sharifi, L., Forge, A., Richardson, G.P., 2012. Hair bundle defects and loss of function in the vestibular end organs of mice lacking the receptor-like inositol lipid phosphatase PTPRQ. J Neurosci 32, 2762-2772.

Goodyear, R.J., Richardson, G.P., 2002. Extracellular matrices associated with the apical surfaces of sensory epithelia in the inner ear: molecular and structural diversity. J Neurobiol 53, 212-227.

Johnson, K.R., Longo-Guess, C.M., Gagnon, L.H., 2012. Mutations of the mouse ELMO domain containing 1 gene (Elmod1) link small GTPase signaling to actin cytoskeleton dynamics in hair cell stereocilia. PLoS One 7, e36074.

Knipper, M., Claussen, C., Ruttiger, L., Zimmermann, U., Lullmann-Rauch, R., Eskelinen, E.L., Schroder, J., Schwake, M., Saftig, P., 2006. Deafness in LIMP2-deficient mice due to early loss of the potassium channel KCNQ1/KCNE1 in marginal cells of the stria vascularis. The Journal of physiology 576, 73-86.

Lee, S.I., Conrad, T., Jones, S.M., Lagziel, A., Starost, M.F., Belyantseva, I.A., Friedman, T.B., Morell, R.J., 2013. A null mutation of mouse Kcna10 causes significant vestibular and mild hearing dysfunction. Hear Res 300, 1-9.

Lubka-Pathak, M., Shah, A.A., Gallozzi, M., Muller, M., Zimmermann, U., Lowenheim, H., Pfister, M., Knipper, M., Blin, N., Schimmang, T., 2011. Altered expression of securin (Pttg1) and serpina3n in the auditory system of hearing-impaired Tff3-deficient mice. Cellular and molecular life sciences : CMLS 68, 2739-2749.

Perrin, B.J., Sonnemann, K.J., Ervasti, J.M., 2010. beta-actin and gamma-actin are each dispensable for auditory hair cell development but required for Stereocilia maintenance. PLoS Genet 6, e1001158.

Pingault, V., Faubert, E., Baral, V., Gherbi, S., Loundon, N., Couloigner, V., Denoyelle, F., Noel-Petroff, N., Ducou Le Pointe, H., Elmaleh-Berges, M., Bondurand, N., Marlin, S., 2015. SOX10 mutations mimic isolated hearing loss. Clinical genetics 88, 352-359.

Schrauwen, I., Helfmann, S., Inagaki, A., Predoehl, F., Tabatabaiefar, M.A., Picher, M.M., Sommen, M., Zazo Seco, C., Oostrik, J., Kremer, H., Dheedene, A., Claes, C., Fransen, E., Chaleshtori, M.H., Coucke, P., Lee, A., Moser, T., Van Camp, G., 2012. A mutation in CABP2, expressed in cochlear hair cells, causes autosomal-recessive hearing impairment. Am J Hum Genet 91, 636-645.

Shabbir, M.I., Ahmed, Z.M., Khan, S.Y., Riazuddin, S., Waryah, A.M., Khan, S.N., Camps, R.D., Ghosh, M., Kabra, M., Belyantseva, I.A., Friedman, T.B., Riazuddin, S., 2006. Mutations of human TMHS cause recessively inherited non-syndromic hearing loss. Journal of medical genetics 43, 634-640.

Somma, G., Alger, H.M., McGuire, R.M., Kretlow, J.D., Ruiz, F.R., Yatsenko, S.A., Stankiewicz, P., Harrison, W., Funk, E., Bergamaschi, A., Oghalai, J.S., Mikos, A.G., Overbeek, P.A., Pereira, F.A., 2012. Head bobber: an insertional mutation causes inner ear defects, hyperactive circling, and deafness. J Assoc Res Otolaryngol 13, 335-349.

Taylor, R., Bullen, A., Johnson, S.L., Grimm-Gunter, E.M., Rivero, F., Marcotti, W., Forge, A., Daudet, N., 2015. Absence of plastin 1 causes abnormal maintenance of hair cell stereocilia and a moderate form of hearing loss in mice. Hum Mol Genet 24, 37-49.

Tong, B., Hornak, A.J., Maison, S.F., Ohlemiller, K.K., Liberman, M.C., Simmons, D.D., 2016. Oncomodulin, an EF-Hand Ca2+ Buffer, Is Critical for Maintaining Cochlear Function in Mice. J Neurosci 36, 1631-1635.

Vuckovic, D., Dawson, S., Scheffer, D.I., Rantanen, T., Morgan, A., Di Stazio, M., Vozzi, D., Nutile, T., Concas, M.P., Biino, G., Nolan, L., Bahl, A., Loukola, A., Viljanen, A., Davis, A., Ciullo, M., Corey, D.P., Pirastu, M., Gasparini, P., Girotto, G., 2015. Genome-wide association analysis on normal hearing function identifies PCDH20 and SLC28A3 as candidates for hearing function and loss. Hum Mol Genet 24, 5655-5664.

Yamamoto, N., Mutai, H., Namba, K., Morita, N., Masuda, S., Nishi, Y., Nakano, A., Masuda, S., Fujioka, M., Kaga, K., Ogawa, K., Matsunaga, T., 2017. Prevalence of TECTA mutation in patients with mid-frequency sensorineural hearing loss. Orphanet journal of rare diseas
